# “Playing the beat”: Occurrence of Bio-duck calls in Santos Basin (Brazil) reveals a complex acoustic behaviour for the Antarctic minke whale (*Balaenoptera bonaerensis*)

**DOI:** 10.1371/journal.pone.0255868

**Published:** 2022-09-15

**Authors:** Marcos R. Rossi-Santos, Diego Filun, William Soares-Filho, Alexandre D. Paro, Leonardo L. Wedekin

**Affiliations:** 1 Laboratório de Ecologia Acústica e Comportamento Animal/ Acoustic Ecology and Animal Behaviour Laboratory, Centro de Ciências Agrarias, Ambientais e Biológicas, Universidade Federal do Recôncavo da Bahia, Cruz das Almas, Bahia, Brazil; 2 Projeto de Monitoramento de Cetáceos da Bacia de Santos- Petrobras/ Cetacean Monitoring Project in Santos Basin, Socioambiental Consultores Associados, Florianópolis, Santa Catarina, Brazil; 3 Ocean Acoustics Lab, Alfred Wegener Institute, Helmholtz Centre for Polar and Marine Research, Bremerhaven, Germany; 4 Projeto de Monitoramento da Paisagem Acústica Submarina da Bacia de Santos- Petrobras/ Underwater Soundscape Monitoring Project in Santos Basin, Instituto de Pesquisas da Marinha, Rio de Janeiro, Rio de Janeiro, Brazil; 5 Marine Biotechnology Program, Instituto de Estudo do Mar Almirante Paulo Moreira, Arraial do Cabo, Rio de Janeiro, Brazil; Wildlife Conservation Society Canada, CANADA

## Abstract

The Antarctic minke whale (*Balaenoptera bonaerensis*) (AMW) is one of the smallest species among baleen whales, occurring in the southern hemisphere from Antarctica to near the equator, and performing seasonal migrations from polar to tropical waters. Information about (AMW) occurrence in the winter breeding grounds is scarce, mostly coming from old records from whaling stations before the 1960’s international moratorium, such as Costinha Station in Northeastern Brazil (6° S / 34° W) and some sightings from few dedicated visual surveys. Acoustic methods can provide important data on the occurrence and distribution of migratory species. This work describes the occurrence of the Antarctic minke whale through acoustic detections of their “Bioduck” vocalisations in the Santos Basin, South-Southeastern Brazil (22° and 28° S / 42° and 48° W). Data was recorded between November 12 and December 19, 2015. AMW calls were detected for 12 days. We detected and classified 9 different Bio-duck calls in Brazilian coastal waters, evidencing a highly diverse acoustic behavior for the minke whale breeding ground. This is the first attempt to describe the acoustic diversity of AMW vocalizations in lower latitudes, contributing important information for future conservation efforts and management of AMW populations and their habitat. Therefore, our study presents the foremost acoustic evidence of the Antarctic minke whale in Brazilian coastal waters.

## Introduction

Marine mammals are key species in the marine ecosystem, generally belonging to higher trophic levels in the food chain, controlling natural populations, cycling nutrients, and providing food when decomposing at the oceanic bottom [1]. For some cetaceans, such as baleen whales, acoustic calls play an important role during foraging and breeding behaviours and have been extensively studied for some species such as the humpback whale (*Megaptera novaeangliae*) [2, 3], the fin whale (*Balaenoptera physalus*) [4, 5], and the blue whale (*Balaenoptera musculus*) [6, 7]. For other whale species, nevertheless, the acoustic ecology remains scarcely understood. Whale vocalisations can show patterns of occurrence, breeding behaviour, movement, and seasonality of a species within a certain area [8–11] and even geographic differences in acoustic repertoire between different areas [12–15].

The Antarctic minke whale (*Balaenoptera bonaerensis*) (AMW) occurs in the southern hemisphere from Antarctica to near the equator (10° S), performing seasonal offshore migrations from polar to tropical waters, like other whale species [16, 17], yet seasonal occurrence, distribution, migration patterns and population structures of AMWs are poorly understood [18].

Notwithstanding, information on AMWs distribution in the South Atlantic Ocean is scarce, restricted to whaling station reports along the 20^th^ century, such as Durban, South Africa (29° 53’ S, 31° 03’ E) from 1968 to 1982 [19] and Costinha, Paraiba state, Northeastern Brazilian coast (6° 53′ S, 34° 52′ W), from 1904 to 1985 [20–23]. More recent information, collected during visual surveys, describes the presence of AMWs during autumn-spring, as one of the most observed species, being sighted only in offshore waters (from 200-4675m) [17, 24–26].

With great technological advances in recent decades, autonomous passive acoustic methods have been considered as an efficient non-intrusive method to study and monitor cetacean ecology and occurrence along an ocean basin [18, 27–29]. In this sense, gliders have been used as an autonomous platform in distinct passive acoustic monitoring, expanding the exploration of less accessible marine habitats, such as deep waters, either in polar or tropical regions of the globe [30, 31] and successfully recording low frequency sounds from baleen whales [32].

This study presents the first acoustic evidence of AMW Bio-duck calls in Brazil, utilizing advanced passive acoustic methods (SeaGlider). The Bio-duck sound was attributed to AMWs in 2014 [33], after more than five decades of unknown “mysterious” recordings in the Southern Ocean. AMW calls have been recorded at high and low latitudes with similar seasonal patterns of occurrence. They are detected during autumn to late spring in different distant locations throughout the southern hemisphere [28, 29].

Species specific whale sounds have been utilized to describe occurrence and distribution patterns for migratory species, highlighting the annual cycle that results in different acoustic environments [2, 27, 34, 35].

The goal of this work is to describe the AMW Bio-duck calls, detected in a potential breeding ground area, Santos Basin (Brazil), using data collected with a SeaGlider. Our study contributes information about occurrence of AMWs in the western South Atlantic Ocean and provides new insights on the acoustic behaviour of this species.

## Methods

### Study area

**Fig 1:**
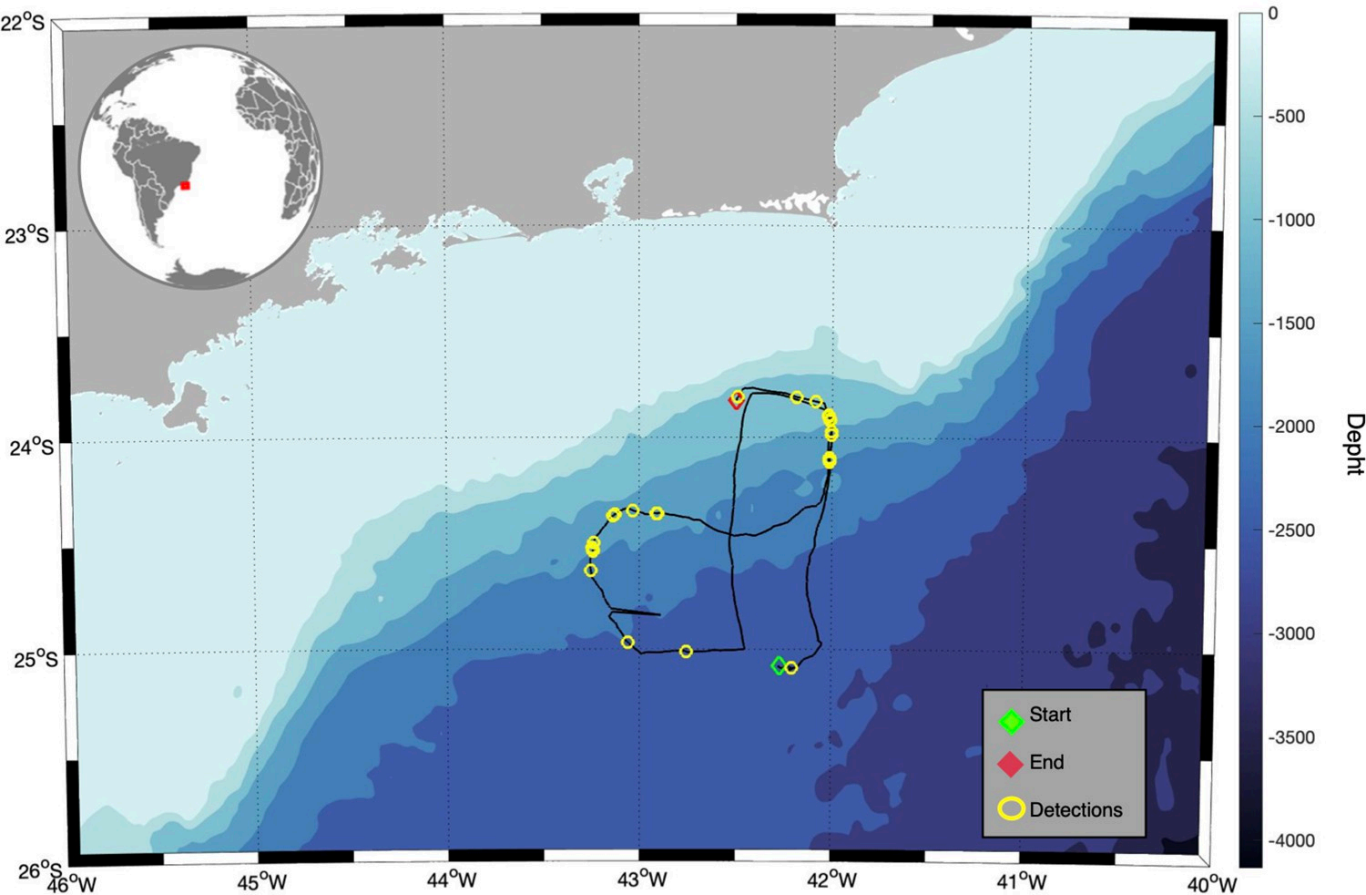
Map of the study area, the Santos Basin, showing the glider track on Brazilian offshore waters between November 12, 2015 (green marker) until December 19, 2015 (red marker). Yellow dots show the positions where Antarctic Minke Whales Bio-duck calls were detected.

The Santos Basin is situated off the south and southeast of the Brazilian coast, between 22° and 28° S and 42° and 48° W, in the western South Atlantic Ocean (Fig 1). This basin presents a wide continental shelf, extending almost 200 km offshore in some locations, crossing a diverse depth gradient, from the Brazilian coastline to pelagic waters over 2.500 m deep, bordered by the Sao Paulo Plateau. The area hosts large oil deposits and multiple fishery resources [36, 37].

### Data collection

The data presented was collected by the Underwater Acoustic Soundscape Monitoring Project (PMPAS-BS, in the Portuguese acronym), according to the reference term 002/2013, as part of a monitoring program required by Brazil’s federal environmental agency, IBAMA, for the environmental licensing process of the oil production and transport by the Brazilian Oil Company (Petrobras) at the Santos Basin pre-salt province.

Since November 2015, PMPAS collects data in the Santos Basin using different types of methodologies, including a glider, equipped with a custom-designed and in-built passive acoustic recording system. The SeaGlider® (Kongsberg Maritime) is designed for continuous, long-term measurements of oceanographic parameters. In this study, the SeaGlider was equipped with a recording system, composed by a HT-92-WB hydrophone, from High Tech Inc., sensitivity: -165 dB re 1V/ μPa, amplified by 25 dB, recorded at 125 kHz sample rate and with 16-bit resolution. It was programmed to record one-hour data every one hour (1/2 duty cycle). Acoustic data is stored in SD cards (up to 500 hours per survey) in the glider and recovered after each survey. The SeaGlider was deployed between 12 November 2015 until 19 December 2015. It was programmed to execute a previous programmed trajectory (S1 Fig) and to perform dives down to 1000 m depth (S2 Fig) with a speed of 25 cm/s (0.5 knot).

### AMW acoustic detection and classification

In this study, a Bio-duck call was defined as multiple series of downsweep pulses clustered, separated by <1 second. The call is characterized by its repetitive nature, consisting of regular downsweeps or pulses in series, with most of the energy located in the 50–300 Hz band, although for signals with higher intensity, harmonics occur up to 1 kHz [33, 35, 38]. The Bio-duck call never occurred alone and it always occurred in repetitive sequences [39].

To detect the occurrence of AMW Bio-duck calls in Brazilian waters, the PAMGuard whistle and moan detector [40, 41] was applied to the acoustic files, as a pre-processing to obtain information on whale signals up to 3 kHz. For this reason, the acoustic data was decimated to 6 kHz and the spectrograms were generated with 1024 FFT points (leading to a window size of 171 milliseconds) and an overlap of 95%. An audio file of a minute length (snipets) was generated when the PAMGuard whistle and moan detector identified Bio-duck calls presence.

**Fig 2:**
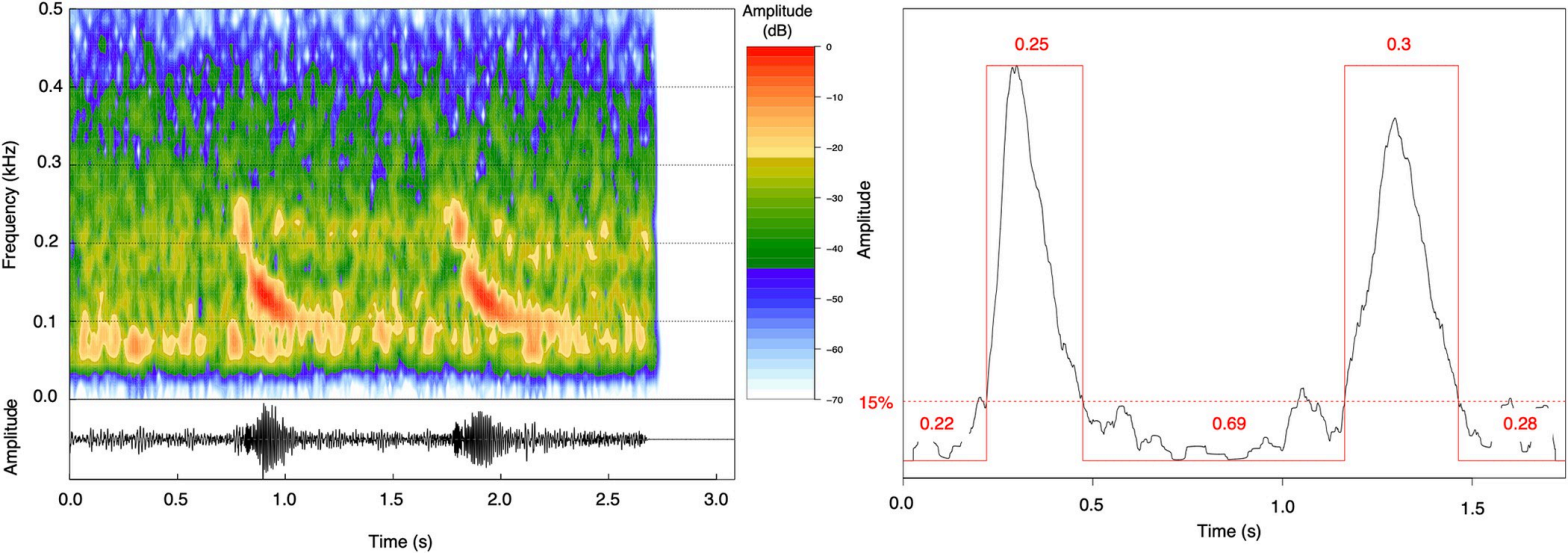
A) Spectrogram of the Bio-duck “A2”, 1125 points FFT, 85% overlap, Hanning window. B) Waveform of the Bio-duck “A2” showing the threshold of the amplitude detector in 15% with an SNR = 18. The figure shows how the detector measures the 1st Pulse, the Inter pulse interval, the 2nd Pulse and the Total duration.

To allow automated feature extraction for call type classification, individual Bio-duck pulses were automatically detected in the 1-minute audio fragments. Spectrograms (1125 points FFT, 85% overlap, Hanning window) of the audio fragments that were extracted from the PAMGuard whistle and moan detector were manually checked in RStudio Version 1.2.5042 with the ‘Seewave’ package [42]. Furthermore, the 1-minute audio files were frequency filtered between 40–500 Hz. For every 1-minute audio fragment, the signal-to-noise ratio (SNR) of Bio-duck calls in the snippet was calculated. In some cases, more Bio-duck pulses were detected in the 1-minute fragments, then, the one with the highest SNR was selected for further processing. To define SNR, for each call, background noise was measured during 0.6 seconds prior to the detected call event in the frequency bands between 40–500 Hz. The signal value was measured during 1.1 seconds after the time point of background noise measurement. The beginning and end of each Bio-duck call in the 1-minute fragment was designated manually. Then, an amplitude filter of 20 dB was applied to increase the SNR of the Bio-duck pulse train spectrum. Only Bio-duck calls with a resulting SNR that was sufficiently high, were selected for further processing using the pulse detector. The pulse detector was used to automatically extract call characteristics from each Bio-duck call for the classification. The automated pulse detector was designed to work using the low frequency downswept component present in all Bio-duck calls. Bio-duck pulse detection thresholds were custom-defined based on the individual Bio-duck call SNR (SNR = 20*log10(rms(signal)/rms(noise))). For acoustic detections with a SNR<5 dB we applied a detector threshold = 50, for detection with SNR>5 dB and SNR<10 dB a threshold = 30, with SNR>10 dB and SNR<15 dB threshold = 20, with SNR>15 dB and SNR<20 dB threshold = 15 and with an SNR>20 dB a threshold = 12 (Fig 2). To reduce false positives of other pulsed signals, only detections of pulses with a duration within the range of 0.05–0.33 seconds (which were the known minimal and maximal durations of AMW pulses), were included as part of the Bio-duck pulse sequence (values measured in this study—Table 2).

For a final classification of the different AMW calls detected in our data set, we used the detections to extract different variables with a custom-built algorithm in RStudio Version 1.2.5042, using the R packages ‘Seewave’ [42, 43] and ‘warbleR’ [44].

**Fig 3:**
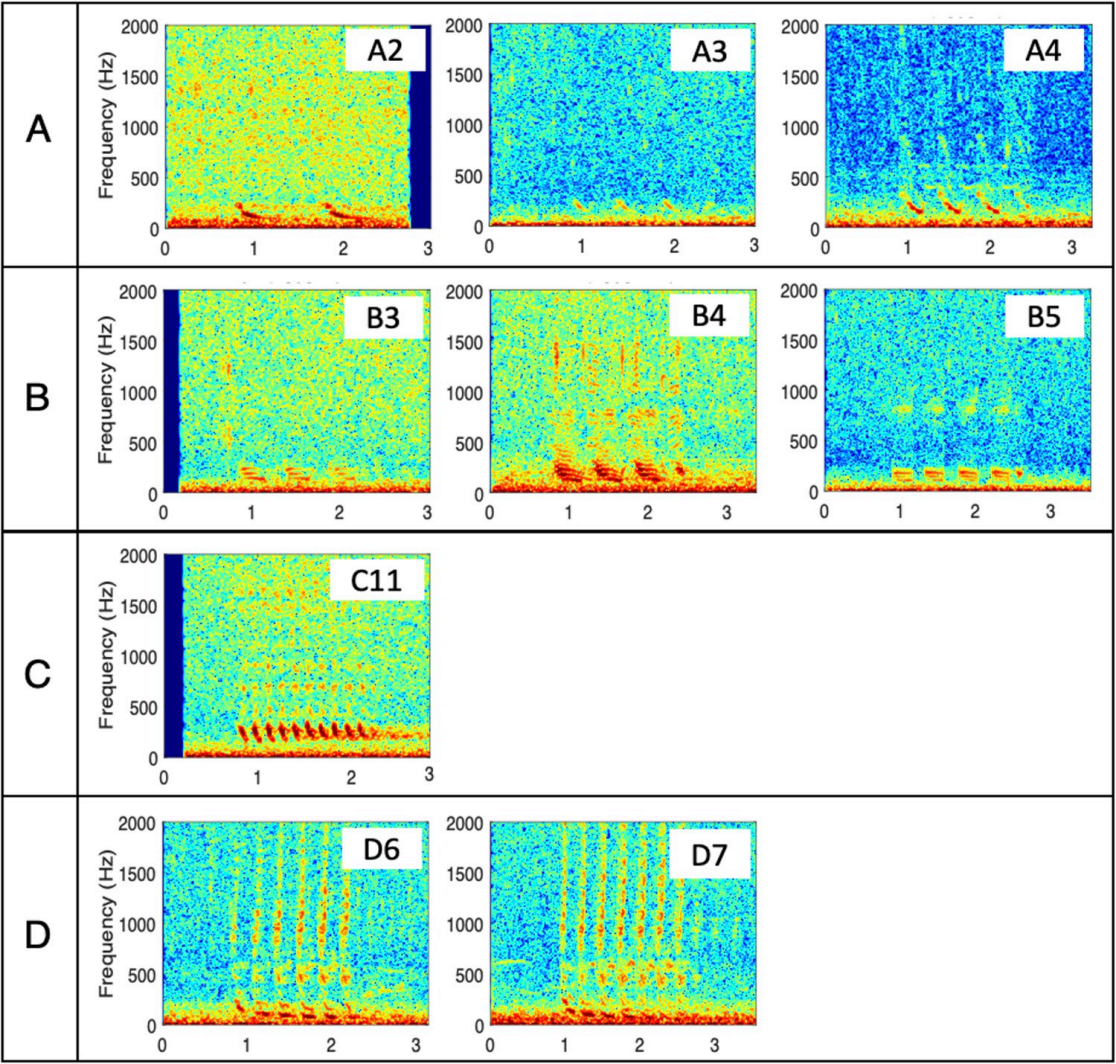
Spectrograms of different Bio-duck calls of the Antarctic minke whale detected in the Santos Basin, Brazilian offshore waters. FFT = 512, overlap = 95%, Time expressed in seconds and frequency scale in Hz.

Parameters that were automatically extracted by the algorithm included the number of pulses (NP), total duration (TD), average duration pulse (average duration of all the pulses that constitute a Bio-duck call) (AvDP), average inter-pulse interval (AvIPI), duration first pulse (DFP), duration last pulse (DLP) and peak frequency (PF) (Table 2). The algorithm is based on an amplitude detector capable to automatically extract and calculate different measurements from the waveform of AMW Bio-duck calls between 40–450 Hz. Although the different types of Bio-duck can have harmonics up to 2,000 Hz (Fig 3), these are not a reliable feature as their presence and quality depends on different factors such as, for example, proximity of the animal to the hydrophone, ambient noise levels and call directionality. Only the fundamental frequency of the downswept pulses was included in the measurements as a robust frequency parameter.

We separated the different Bio-duck calls using cluster analysis based on the calculated acoustic parameters in order to optimize the classification (see Table 2). A Euclidean method was used to calculate distances between the different clusters. To determine the number of clusters (*k*) we used the ‘NbClust’ package [45]. This automatically calculates and provides 30 different indices for determining an appropriate number of *k* from different results obtained by varying all combinations for number of clusters, distance measures and clustering methods.

Different parameters extracted from each Bio-duck call type were used to perform a hierarchical agglomerative cluster analysis to generate unsupervised call type groups. To perform the automatic classification analysis, we used values extracted from 30 samples for each type of Bio-duck detected. From type B3, due to the small number of samples, we were able to use only 14 calls.

## Results

Between November 12 to December 19, 2015, the SeaGlider covered approximately 725 km and collected 262 hours of acoustic data during 187 glider dives. AMW calls were detected along the transect for 12 days (Table 1). Manual analyses confirmed that detections were AMW Bio-duck calls, based on the literature description [33, 35, 38, 46].

**Table 1:** Antarctic minke whale Bio-duck call encounters in Brazilian offshore waters.

Date	Hour start	Hour end	Latitude S°	Longitude W°	Glider depth (m)	Bio-duck types
12.11.2015	13:49:00	13:54:41	-23.80815506	-42.48846817	112.98	D6
14.11.2015	08:26:00	08:43:40	-23.80920029	-42.18441772	1.30	D6
14.11.2015	15:47:00	16:04:40	-23.82978058	-42.08476257	279.75	D6
15.11.2015	03:16:00	03:49:47	-23.89512062	-42.02005386	130.18	D6
15.11.2015	03:50:47	04:14:46	-23.89885521	-42.01930618	52.63	C11
15.11.2015	05:46:00	06:21:21	-23.9147644	-42.01307678	345.10	D6
15.11.2015	06:23:21	06:57:20	-23.91983223	-42.01108932	118.74	A4
15.11.2015	11:50:00	12:21:07	-23.97163963	-42.00485611	876.99	A4
15.11.2015	13:31:05	13:41:05	-23.98914909	-42.0019455	108.62	A4
16.11.2015	00:12:00	00:43:19	-24.09622574	-42.01313782	947.00	B3
16.11.2015	00:45:19	01:17:18	-24.10261917	-42.01353836	874.85	D6
16.11.2015	01:19:18	01:51:17	-24.10923958	-42.01395416	645.67	B4
16.11.2015	01:53:17	02:17:16	-24.11581612	-42.01436615	435.60	B3
22.11.2015	07:37:27	08:09:26	-24.35966492	-42.90554047	129.98	B3
22.11.2015	08:11:26	08:23:26	-24.35882759	-42.90994263	177.80	D6
23.11.2015	09:39:00	10:08:33	-24.34605026	-43.03238297	761.20	D6
24.11.2015	13:26:00	13:38:07	-24.36293983	-43.12480164	150.02	B5
24.11.2015	15:16:16	15:44:15	-24.37080193	-43.13537979	121.74	D7
25.11.2015	17:30:52	17:52:51	-24.49822235	-43.2351265	525.01	D6
25.11.2015	21:12:00	21:43:14	-24.52141762	-43.24263382	1.28	D6
25.11.2015	23:13:16	23:43:15	-24.53253174	-43.2402153	589.22	A2
25.11.2015	23:45:15	23:50:15	-24.5354538	-43.23957825	198.02	A2
26.11.2015	00:01:15	00:11:14	-24.53692055	-43.23926163	6.73	A4
26.11.2015	15:11:26	02:39:00	-24.63012505	-43.2525177	133.27	A4
29.11.2015	22:37:30	23:44:49	-24.96931648	-43.05936813	409.64	A3
01.12.2015	19:34:00	19:41:03	-25.01185799	-42.75591278	546.45	A4
17.12.2015	14:27:00	14:44:00	-25.08859062	-42.20511627	4.25	A4

### Description of vocalizations

The vocal classes, or groups, were named according to acoustic properties extracted from Bio-duck calls detected in Brazilian waters based on an automatic classification (Table 2) and indicated in capital letters followed by a number. The letters correspond to the groups of clusters identified based on the previously extracted acoustic measurements. The number corresponds of downswept pulses present in a classified Bio-duck call (Fig 2). Call types were qualitatively named, to maintain consistency between naming schemes and sound structures. For the automatic measurements we did not consider harmonics, because they varied in relation of the proximity of the animals to the SeaGlider. We do show, however, spectrograms with the full frequency range to distinguish harmonics in the Bio-duck calls detected, going up to 2000 Hz.

**Table 2:** Antarctic minke whale Bio-duck call extracted measurements with the amplitude detector. All time variables measured in seconds *(s)*, *(n)* = no of samples, Peak Frequency measured in Hertz.

Cluster	NP	TD (s)	Av DP (s)	Av IPI (s)	DFP (s)	DLP (s)	PF (Hz)	(*n*)
A	2	1.26±0.3	0.28±0.4	0.7±0.3	0.28±0.2	0.29±0.4	131.8±15	30
A	3	1.3±0.2	0.31±0.3	0.19±0.2	0.32±0.3	0.3±0.2	182.9± 12	30
A	4	1.61±0.2	0.28±0.3	0.17±0.1	0.33±0.1	0.1±0.3	177.4±9	30
B	3	1.04±0.2	0.09±0.2	0.38±0.0	0.08±0.2	0.09±0.2	162.9±16	14
B	4	1.45±0.3	0.19±0.2	0.23±0.0	0.18±0.2	0.09±0.2	181.6±16	30
B	5	1.7±0.3	0.23±0.5	0.14±0.1	0.27±0.2	0.1±0.1	159.8±8	30
C	11	1.45±0.2	0.08±0.3	0.05±0.0	0.07±0.1	0.08±0.3	245.6±10	30
D	6	1.39±0.3	0.14±0.4	0.11±0.2	0.11±0.2	0.11±0.2	116.3±18	30
D	7	1.62±0.3	0.16±0.2	0.08±0.2	0.18±0.1	0.06±0.2	86.3± 10	30

### Bio-duck calls classification

**Fig 4:**
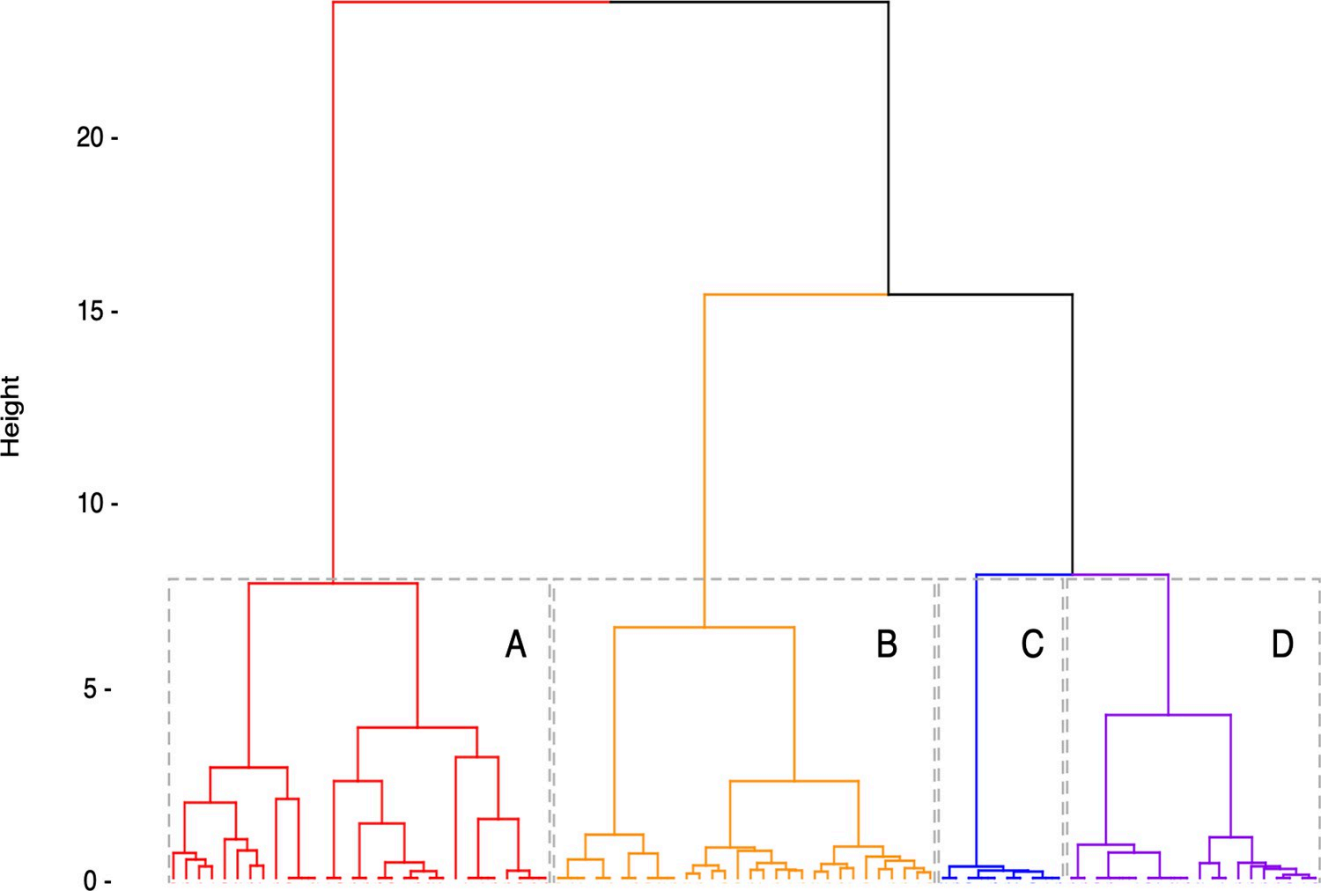
Dendrogram showing results of a hierarchical cluster analysis. Clusters representing four classes of Antarctic minke whale Bio-duck calls. Cluster A corresponds to A2, A3 and A4; Cluster B with B3, B4 and B5 sounds; Cluster C with the C11; and Cluster D with D6 and D7 calls.

Nine different Bio-duck calls (Fig 3) were detected and classified with a hierarchical agglomerative cluster analysis (Fig 4). Every call analyzed contained between 2 and 11 pulses. Duration of a Bio-duck sound ranged between 1.04–1.70 seconds, and the average pulse duration between 0.08–0.31 seconds. On the other hand, the AvIPI ranged between 0.05–0.38 seconds. For all categories, whenever a Bio-duck call increased in the number of pulses, the AvIPI decreased, whereas the total duration increased. The DFP varied from 0.07–0.33 seconds and the DLP between 0.06–0.3 seconds. The downswept component frequency peak was 86.3 to 245.6 Hz (Table 2). The analysis identified 4 different clusters (73%), grouping the “A2”, “A3” and “A4” calls in the cluster A, Bio-ducks “B3”, “B4” and “B5” in the cluster B, and only one call, “C10”, in the cluster C. Finally, the cluster D was composed by two calls: “D6” and “D7” (Fig 4).

### Bio-duck call occurrence

Bio-duck call named “D6” was the most common call detected in the data, being present during 7 different days. Second most common call detected was Bio-duck “A4” evidenced in 4 days. Bio-duck “B4” was detected in 3 different days. Other Bio-duck calls, however, were detected only one day along the glider deployment period analyzed. During the 15/11/2015, three different Bio-duck calls were detected, being the day with most acoustic diversity of Bio-duck call occurrence, from 3:16 am until 1:41 pm. During that day, Bio-duck calls “D6”, “A4” and “C11” were detected. Second day with most acoustic variability was the 25/11/2015 with the presence of two different call types (“D6” and “A2”). The rest of the days with acoustic presence, the type of call detected was repeated throughout the day, and was the only call type detected Table 1.

## Discussion

This work presents 9 different AMW Bio-duck calls found in the Brazilian coast, a presumed low latitude breeding ground for migratory baleen whales in the western South Atlantic Ocean. This provides the highest diversity of acoustic behavior reported for the species so far. The diversity of calls found here, suggests that AMW may produce organized and repetitive sounds, which suits in the description of songs for other whale species. In addition to the variability of sounds produced by AMWs, their vocalizations are produced in repetitive sequences (S3 Fig) just like those of, for example, Antarctic blue whales [47] or fin whales [48–50].

### Bio-duck calls comparison

Regarding acoustic structures, the Bio-duck has been considered a very conspicuous signature call [18, 33, 39, 46]. The most obvious characteristic is that energy is in a frequency band ranging from 50 to 500 Hz [33, 35, 38, 46], although more intense signal harmonics have been observed up to 2000 Hz.

The first study attributing Bio-duck sounds to AMWs, described calls in series between 5–12 pulses, produced in regular sequences with an inter-sequence interval of 3.1 s. Data was collected from a tagged AMW in Wilhelmina Bay, in the Antarctic Peninsula, and they described 3 different Bio-ducks, composed between 3–7 pulses and 3 types of downsweep low-frequencies [33]. Another study in the Antarctic Peninsula [35], using year round acoustic data from a mooring position, described 4 distinct Bio-duck call variants, with one variant having two sub-types. Calls were described in sequences ranging between 4–13 pulses. In addition, this study also indicates the presence of a call type of low-frequency downsweep. Recently [38], described the occurrence of AMW calls in South African waters and Maud Rise, Antarctica. Vocalisations detected in the study presented harmonics up to 2000 Hz and were classified in 3 categories with one category presenting 2 sub-types. Bio-ducks presented sequences varying between 4–10 pulses. In Perth Canyon, western Australia [46], used a time ratio method for detecting the Bio-duck signal, distinguishing two different types of call. One with a low repetition rate (T = 1.6 seconds) and one with approximately doubled period (T = 3.1 seconds).

Based on a simple visual comparison of spectrograms of Bio-ducks detected in other studies the calls described in our study match some of the Bio-ducks previously described in Perth Canyon [46], Western Antarctic Peninsula [33, 35], South Africa and Maud Rise, Antarctica [38]. Similarly, 7 of 9 calls detected in Brazil, classified in different categories, presented harmonics up to 2000 Hz. At first glance, comparing the spectrograms of our study with [38], the shape and structure of the South African Bio-duck C-type resembles category C from this study with the South African Bio-duck being composed of 4 pulses versus 6 and 7 pulses in this study. This information highlights the complex structure of the vocal behaviour produced by AMWs. New classification suggested in this study, indicates that based on different acoustic parameters extracted from Bio-duck calls, these can be classified into different categories. Furthermore, these categories are composed of different calls which are called subtypes and are differentiated according to the number of pulses that make up each call. With the information that AMW Bio-duck calls can be classified into categories, studying the subtypes of sounds could help to better understand the acoustic behaviour of this species, and even know if through acoustics it is possible to identify specific populations.

### Antarctic minke whales in Brazil and similarity with other regions

Comparing present data with other regions, the acoustic occurrence seems to match. In Brazil (24 and 25° S), we recorded Bio-duck calls during November and December. Likewise, Bio-duck calls were recorded in Perth Canyon (32° S), between October and December [46]. In Namibia (20° S), a double peak was recorded between June-August and November-December [51]. In South African waters (34°S) a peak of acoustic activity was described between September-October [38] and in the Juan Fernandez Archipelago, off Chile, (33°S) a year-round acoustic presence was recorded with a peak between May-August [52].

When comparing low and high latitude areas, there appears to be similarities in the acoustic seasonality of AMWs. In the Southern Ocean, specifically in the western Antarctic Peninsula, periods of acoustic activity have been described for AMWs between the months of May-November with peaks during July-October [35]. A similar situation occurs in the Weddell Sea, where the acoustic presence has been described between May-December, with a peak during June-November [28, 35, 39]. The resemblance in seasonality of the acoustic behavior of AMWs between low and high latitudes, suggests that not all individuals migrate to breeding grounds. Part of the population possibly stays in Southern Ocean waters for a longer time than in higher latitudes.

Once distribution and ecological relations of *Balaenoptera* sp. in their breeding grounds are scarcely understood, this study contributes with the recordings of AMW in Santos Basin, western South Atlantic Ocean, between 24 and 25° S, while Perth Canyon, Indian Ocean, lies at 32° S. Furthermore, the presence of Bio-duck calls, indicates that AMW is present on the Brazilian continental slope and in oceanic waters, as suggested by other recent observations [17, 24–26].

The use of the Seaglider showed to be quite efficient for the scarcely investigated Brazilian shelf and Slope. As pointed out before [30, 32], this equipment brings the advantage in terms of the ability to collect recordings at different depths and the possibility to integrate with other sensor data, as well as spatial coverage. Applying this technology, we were able to provide this novel data on AMW in Brazilian waters, presented in this manuscript.

## Conclusion

In summary, our results show that Antarctic minke whales perform a diverse repertoire of Bio-duck calls in oceanic waters off Brazil. The distinctiveness of these type of calls in Santos Basin reinforces the occurrence of this species in the region, which is further south than main ex-whaling areas, where their presence is well documented through historical accounts. Further studies using passive acoustics to detect Bio-duck calls may raise important information about this species seasonality, migratory timing and connections with other feeding and breeding regions of the southern hemisphere.

This study also highlights the importance of further implementation of passive acoustic monitoring in Brazil, nowadays limited to a few initiatives, as requirements for oil and gas seismic and exploitation activities. We strongly suggest that the use of passive acoustic monitoring should be a mandatory routine to study cetacean occurrence, behavior, and potential noise impacts on them, in many other marine exploitation activities, such as port constructions and cargo ship traffic, considered to impact Brazilian coast without any previous assessment [53]. 1 Further investigation with a broader acoustic dataset can elucidate important questions about the minke whale annual cycle, such as migration timing, if they are breeding, foraging or doing both vital activities while in their tropical breeding grounds.

## Supporting information

S1 Fig Scheduled recording scheme for the glider monitoring period (12 November—19 December 2015). Recording periods for each monitoring day (red squares). The glider was programmed to record one hour every one hour (1/2 duty cycle). From 03 December the glider started to have problems to continue with the scheduled duty cycle due to technical problems.
(TIF)

S2 Fig Glider trajectory, highlighting the sections with recording. Includes a dashed line indicating the programmed trajectory. Green and Red squares shown the start and end position, respectively, of the SeaGlider deployment trajectory.
(TIF)

S3 Fig Spectrogram displaying a “Song-like” Bioduck of type B4, produced by the AMW in Santos Basin, Brazil. In this area, BioDuck sounds are produced in repetitive and long sequences, like “songs”, as known for other whale species. Hanning Window 75% overlap, 1145 points FFT.
(TIF)
