# Supplementary figures and images for: “Playing the beat”: Occurrence of Bio-duck calls in Santos Basin (Brazil) reveals a complex acoustic behaviour for the Antarctic minke whale (Balaenoptera bonaerensis)
Source: PLoS One. 2022 Sep 15;17(9):e0255868. doi: 10.1371/journal.pone.0255868 (PMC9477358; doi:10.1371/journal.pone.0255868)

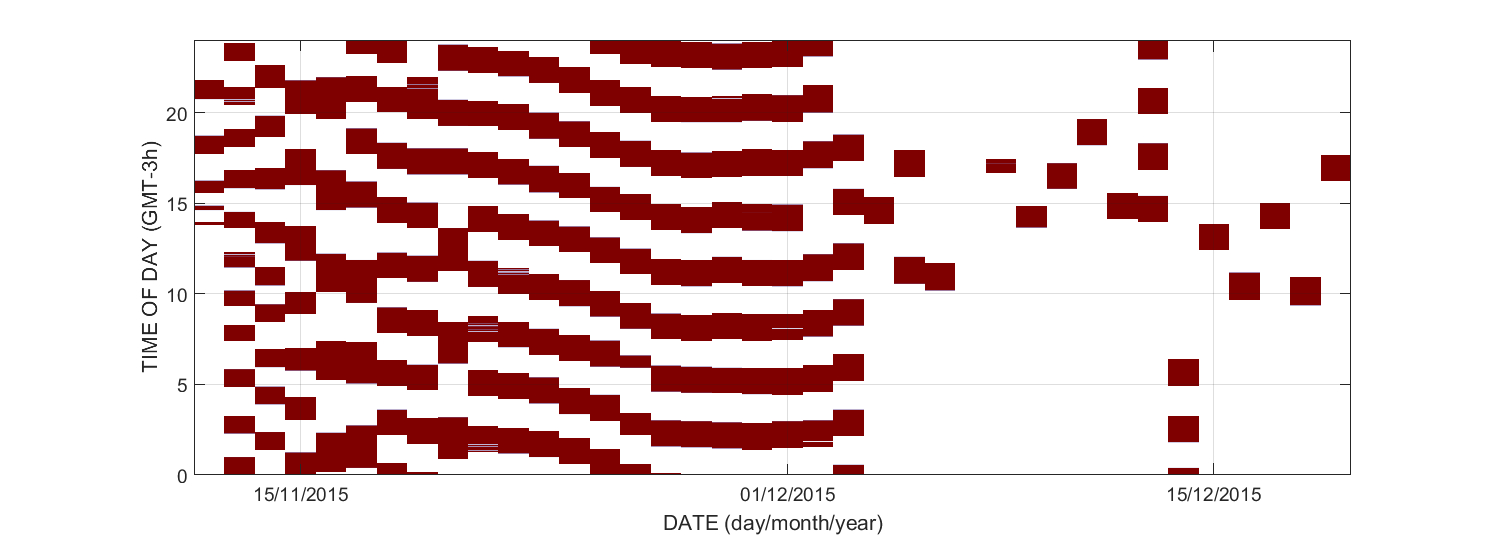

Supplement: S1 Fig — Recording periods for each monitoring day (red squares). The glider was programmed to record one hour every one hour (1/2 duty cycle). From 03 December the glider started to have problems to continue with the scheduled duty cycle due to technical problems. (TIF) [file pone.0255868.s001.tif]

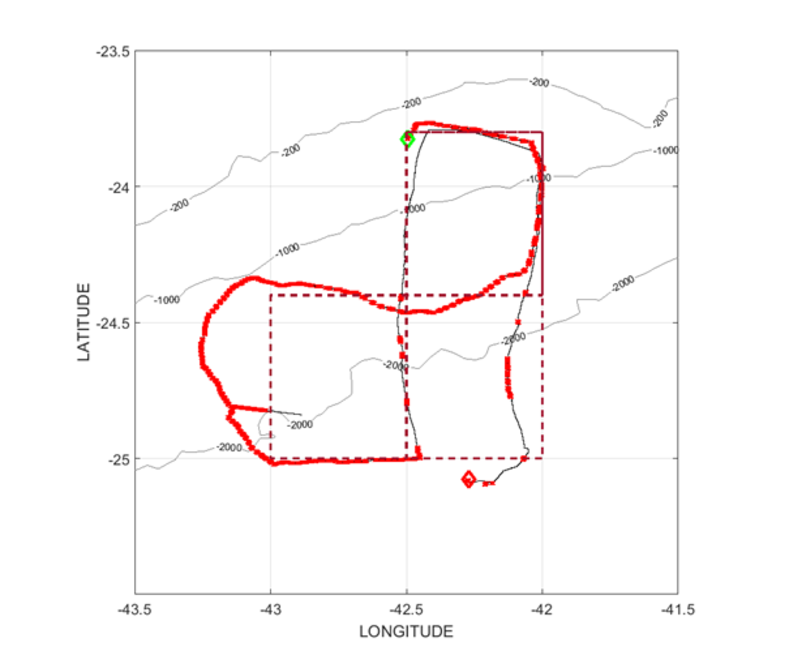

Supplement: S2 Fig — Includes a dashed line indicating the programmed trajectory. Green and Red squares shown the start and end position, respectively, of the SeaGlider deployment trajectory. (TIF) [file pone.0255868.s002.tif]

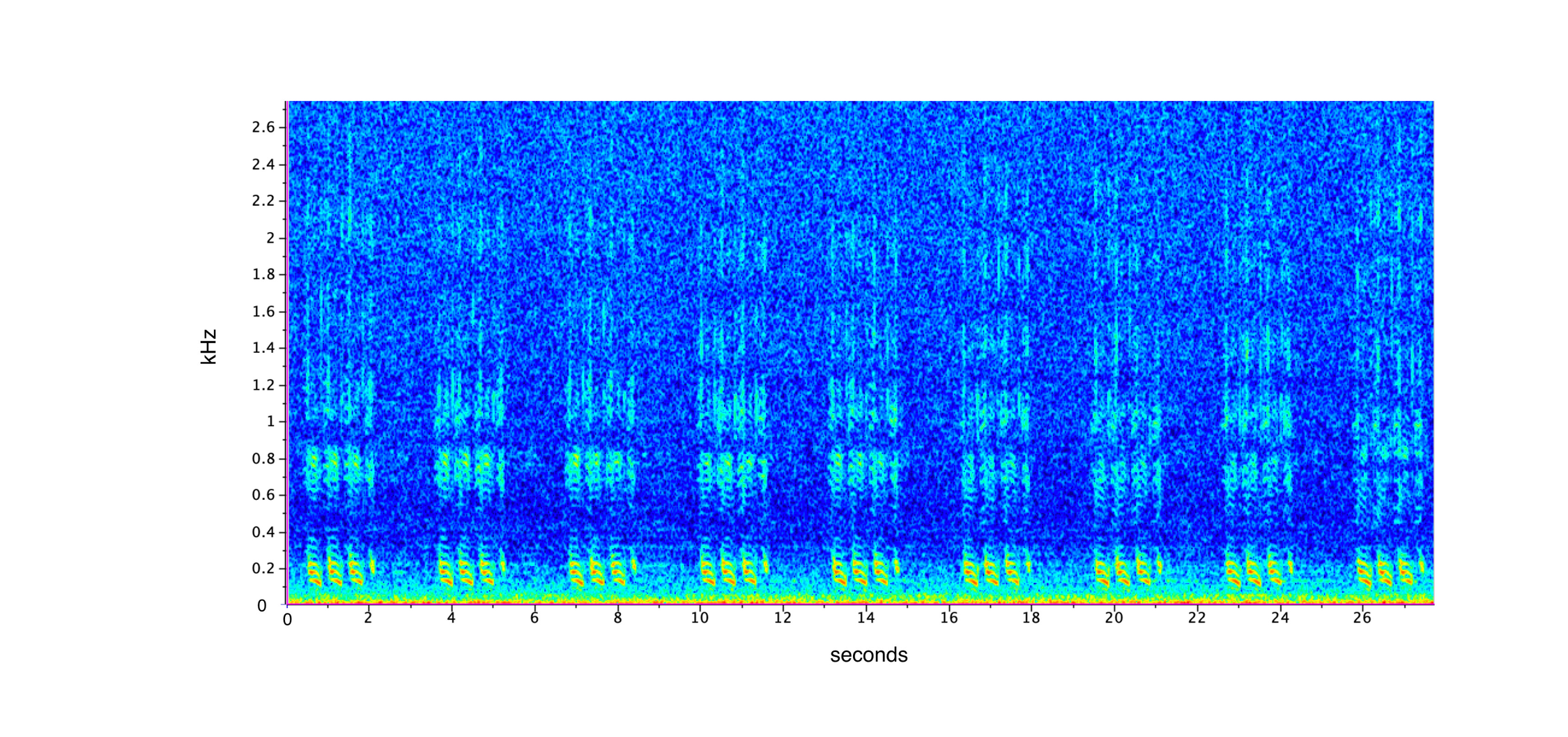

Supplement: S3 Fig — In this area, BioDuck sounds are produced in repetitive and long sequences, like “songs”, as known for other whale species. Hanning Window 75% overlap, 1145 points FFT. (TIF) [file pone.0255868.s003.tif]
